# Multidisciplinary integrated headache care: a prospective 12-month follow-up observational study

**DOI:** 10.1007/s10194-012-0469-y

**Published:** 2012-07-12

**Authors:** Thomas-Martin Wallasch, Peter Kropp

**Affiliations:** 1 Headache Center Berlin at the Sankt Gertrauden Krankenhaus, Berlin, Germany; 2 MEDAS Ostschweiz, Kornhausstr. 3, 9000 St Gallen, Switzerland; 3 Medical Faculty of the University, Institute of Medical Psychology and Medical Sociology, Rostock, Germany

**Keywords:** Integrated, Care, Multidisciplinary treatment program, Outcome study, Headache-related disability, Headache-related quality of life, Chronic headache

## Abstract

This prospective study investigated the effectiveness of a three-tier modularized out- and inpatient multidisciplinary integrated headache care program. *N* = 204 patients with frequent headaches (63 migraine, 11 tension-type headache, 59 migraine + tension-type headache, 68 medication-overuse headache and 3 with other primary headaches) were enrolled. Outcome measures at baseline, 6- and 12-month follow-ups included headache frequency, Migraine Disability Assessment (MIDAS), Hospital Anxiety and Depression Scale (HADS), standardized headache diary and a medication survey. Mean reduction in headache frequency was 5.5 ± 8.5 days/month, *p* < 0.001 at 6 months’ follow-up and 6.9 ± 8.3 days/month, *p* < 0.001 after 1 year. MIDAS decreased from 53.0 ± 60.8 to 37.0 ± 52.4 points, *p* < 0.001 after 6 months and 34.4 ± 53.2 points, *p* < 0.001 at 1 year. 44.0 % patients demonstrated at baseline an increased HAD-score for anxiety and 16.7 % of patients revealed a HAD-score indicating a depression. At the end of treatment statistically significant changes could be observed for anxiety (*p* < 0.001) and depression (*p* < 0.006). The intake frequency of attack-aborting medication decreased from 10.3 ± 7.3 days/month at admission to 4.7 ± 4.1 days/month, *p* < 0.001 after 6 months and reached 3.8 ± 3.5 days/month, *p* < 0.001 after 1 year. At baseline 37.9 % of patients had experience with non-pharmacological treatments and 87.0 % at 12-month follow-up. In conclusion, an integrated headache care program was successfully established. Positive health-related outcomes could be obtained with a multidisciplinary out- and inpatient headache treatment program.

## Introduction

Chronic headache refers to a heterogeneous group of headache disorders causing severe burden of disease on society and involves high costs in healthcare systems. Chronic headache reduces quality of life, decreases social and job functioning and increases utilization of headache-related services. Accordingly, headache disorders are amongst the top ten causes of disability [1, 2]. Increasing headache frequency often leads to frequent intake of triptans and analgesics resulting in medication overuse headache (MOH), which is a complication of headache treatment and is characterized by a headache occurring on 15 or more days per month for more than 3 months [3, 4]. However, medication overuse can cause chronic headache in patients suffering from a primary headache disorder [5]. In clinical practice, chronic headache is mainly represented by these three headache disorders, which have major significance for public health because they are common and responsible for almost all headache-related burden [6].

However, chronic headache is difficult to treat. Despite advances in acute and prophylactic treatment of primary headaches, many headache sufferers remain misdiagnosed and undertreated [7–9]. In general, standard care therapy for headache patients is provided by general practitioners and neurologists in private practice. But most of primary care physicians are not specialized in headache care. Moreover, structured concepts of headache treatment using effectively primary, secondary and tertiary health care systems do not exist in many countries [6]. Additionally, overprotected or unrestricted access to headache specialists induces probably further complications, which will lead to higher costs or blocking of restricted personal resources for the therapy of difficult-to-treat headache patients. These problems result in ineffective headache diagnosis and treatment, which leads headache patients to repeated consultations of different disciplines, expenditure on alternative therapies and unnecessary hospitalizations. Patients with chronic headache utilize more health care resources and claim twice as much medication compared with patients with other diseases resulting in high indirect and direct costs [2, 10].

Some multidisciplinary headache programs have already been established for patients with frequent refractory headaches [11–25], but documentation of organization, as well as published outcome and follow-up data for periods longer than 3 to 6 months, is still sparse. Although there is sufficient evidence that mood and affective disorders affect the outcome in chronic headache patients, most of the available studies lack data on psychiatric comorbidities [26, 27]. In order to overcome these problems in daily practice, we developed a multidisciplinary headache treatment program in a tertiary headache center in Berlin, Germany, that entails a comprehensive assessment including a headache diagnosis according to ICDH-II criteria [28], screening for psychiatric comorbidity and musculoskeletal disorders and provides treatment according to clinical guidelines [29, 30]. Our integrated headache care program follows the recommendations of a three-tier interdisciplinary system [6]. The multidisciplinary headache treatment program started in 2006 in cooperation with selected health insurance companies and the University Hospital in Essen, Germany, which initiated a similar integrated headache care program in 2005 [24, 31]. Here, we report prospectively collected baseline, 6- and 12-month follow-up data from frequent migraine, TTH and MOH and/or difficult-to-treat chronic headache patients. All questionnaires were administered on a pocket PC. Follow-up focused on changes in headache frequency and headache disability using MIDAS Questionnaire [32, 33]. Psychiatric comorbidity of anxiety and depression was documented by HAD-Scale [34]. Additionally, the intake frequency and an overuse of attack-aborting medications (analgesics and triptans), use of prophylactic headache medication and non-pharmacological treatments according the recommendations of the German Headache and Migraine Society [29, 30] were further follow-up parameters. Primary and secondary responder rates and outcomes in modules are reported separately.

## Methods

### Organization

The Headache Center Berlin (HCB) was inaugurated in 2006 as a tertiary headache clinic and provided an outpatient and day clinic service for patients with chronic frequent and/or difficult-to-treat headaches. Inpatient treatment facilities (five beds) for patients with medication overuse and severe psychiatric comorbidity were available in cooperation with the Sankt Gertrauden Hospital Berlin, Germany. The main uptake area was Berlin and the north-eastern districts of Germany with a population of 7,600,000. The capacity was 750 new patients per year. The staff in the Headache center consisted of one full-time senior headache specialist supplemented by one full-time junior doctor, two part-time behavioural psychologists and two physical and sports therapists, one full-time nurse and one secretary, with consultants from psychiatry, otorhinolaryngology, ophthalmology, internal medicine and dentistry. Patients had to be referred by health insurances or neurologists. The HCB cooperated with a network of headache specialists (secondary care). All network partners were connected with the HCB by a specifically dedicated computer documentation system for online documentation and collection of patient data [35]. At the HCB, European Headache Federation guidelines for the organization of headache clinics were implemented [11].

### Study design

This was a prospective observational, non-randomized study reporting the outcome in patients suffering from high frequency and/or difficult-to-treat headaches following the integrated headache care program of the HCB. All patients gave written informed consent prior to participation. The project was approved by the local Ethics Committee. Adult patients (≥18 years) with headache diagnosis of migraine, tension-type headache (TTH), combination headache and/or MOH according to ICHD-II criteria [28] referred to the HCB for the first time between 3/2009 and 9/2009 were consecutively enrolled.

### Procedures

Patients were referred by physicians or specialists when headache treatment failed. In addition, health insurance companies identified eligible patients on the basis of inpatient data, sick leave or records of prescribed medication. Prior to the first visit, all participants kept a standardized headache diary (http://www.dmkg.de/dmkg/sites/default/files/ks_kal.pdf) for at least 4 weeks. Initial sessions consisted of an individual comprehensive assessment by a neurologist, a psychologist and a physical therapist taking one-hour face-to-face contact for each. If necessary, additional diagnostic tests (imaging, blood test, etc.) and consultations with further disciplines were performed. A headache diagnosis was made according to ICHD-II criteria [28]. The psychologist obtained information about the patient’s level of stress, emotional well-being, job satisfaction, life events and possible psychological headache triggers. Mental disorders were diagnosed clinically by a standardized interview using DIPS methodology [36] and classified based on ICD-10 criteria [28]. A physical therapist examined posture and muscle function and instructed patients in active exercises and in the identification and avoidance of possible muscular stress factors. Passive treatment strategies, such as massage, were not performed. Additionally, patients completed several questionnaires. All questionnaires were administered on a pocket PC using AC-STB software from Akkaya company, Cologne, Germany; [35]. The Migraine Disability Assessment (MIDAS) Questionnaire [32, 33] was developed to assess headache-related disability. Headache-related quality of life was assessed using the 12-item Short-Form Health Survey (SF12 [37], German version: [38]. The SF12 contains 2 subscales of functioning (“physical”/“psychological”). The Hospital Anxiety and Depression Scale (HADS, (German: [36]) was used to assess depression and anxiety. The HADS is recommended for patients with somatic problems [39]. Subsequent to the assessment, both team and patient met in a pain conference and made a joint decision about further treatment, which was realized in co-operation between the HCB and the corresponding network headache specialists. The managed care system entailed a modularized treatment protocol. Patients were assigned to one of three treatment modules taking into consideration headache frequency, medication overuse and psychiatric comorbidity.

*Module 1:* Moderate chronicity—Patients with a headache frequency between 6 and less than 10 days/month and less than 10 days with analgesics/triptans intake were assigned to this module. Treatment included education and patient self-management for preventing headache episodes. If necessary, medication was optimized and patients were treated near their place of residence by the complementary specialists. A joint patient file, medical letters and follow-up documentations were transmitted using the AC-STB software [35].

*Module 2:* Severe chronicity—Patients with more than 10 headache days/month and more than 10 days with analgesics/triptans intake were assigned to this module. Patients received module 1 treatment and took part in a multidisciplinary treatment program (MTP) at the HCB. MTP consisted of a maximum of 12 additional outpatient sessions on five consecutive days from 9.00 am to 4.00 pm with a break of 1 h (30 h of therapy). The program entailed group sessions of maximum 12 patients and included individual appointments with the neurologist, psychologist and the physical therapist if needed. The senior neurologist provided headache education (90 min per day) focussing on informing patients about etiology and pathophysiology and the symptoms of primary headaches and MOH, medical and non-pharmacological treatment options, and correct use, efficacy and possible adverse effects of acute and prophylactic medication. The psychologist provided cognitive-behavioural pain management (90 min per day). Psychological group sessions focused on recommendations of lifestyle for headache sufferers, and discussion of individual headache concepts, individual styles of coping with headache, avoidance of headache triggers and stress management. Furthermore, patients performed progressive muscle relaxation therapy (PMR; 60 min per day). All patients received a CD to enable them to practice PMR at home daily. The physical training comprised endurance sport, physical therapy and Nordic walking (60 min per day). One important component of the behavioural therapy concept was to motivate the patient at all levels during the treatment program to immediately integrate the newly gained knowledge and non-drug treatment techniques in daily life.

*Module 3:* Severe chronicity with additional problems—Patients with more than 15 headache days/month and more than 15 days with analgesics/triptans intake and severe psychiatric comorbidity or psycho-social problems, which made withdrawal at home impossible, were assigned to this module. They received module 1 and 2 treatment and apart from participating in the MTP, they were hospitalized for a maximum of 5 days and underwent drug withdrawal. Treatment entailed initiating adequate acute and prophylactic drug management.

### Baseline and follow-up data

The baseline data and the follow-up survey at 12 months were performed by face-to-face interviews with the physicians and medical staff of the HCB, while 6-month data were collected by the network headache specialists of the integrated care system. These data were administered using AC-STB software from Akkaya company, Köln, Germany [35]. Demographic and personal data were obtained at baseline as well as attack-aborting and prophylactic medication and use of non-pharmacological treatments. The course of these data and the headache frequency during the one-year follow-up were reported by the patients on the basis of prospective headache diaries. Burden of disease was measured using the MIDAS Questionnaire. Further key areas covered in the instruments included measurement of depression and anxiety using the HAD-Scale. All these data were documented by the patients on a hand-held PC using AC-STB software. Information concerning psychosocial status was obtained on a voluntary basis.

### Data analyses

Depending on the type of outcome variables, differences of data between measurement points (baseline—6 months—12 months) were computed either with Student *t* test for continuous variables or Mann–Whitney *U* test when variables were not normally distributed. We used Chi^2^ test for comparisons of categorical variables. A *p* value of 0.05 was considered significant. For better tracking statistical data analyses we labeled also degrees of freedom (*df*) within the text. All analyses were performed using IBM, Predictive Analytics SoftWare (PASW), by SPSS, Version 19.0.0.

## Results

### Cohort and baseline data

A cohort of 337 chronic headache sufferers was consecutively referred to the HCB between 3/2009 and 9/2009, of whom 213 were qualified for participation in the multidisciplinary integrated care program. 101 patients could not be included because their insurances did not pay for them; they received standard care. 23 patients had no chronic headache disorders as defined in the treatment protocol for modules 1 to 3 or had headache diagnoses other than migraine, TTH or MOH as defined as chronic headache disorder on more than 15 days/month caused by medication intake of attack aborting drugs in patients suffering from a primary headache disorder. Nine patients were excluded (*n* = 5 were lost for personal reasons, *n* = 3 moved away and 2 became pregnant and did not want to continue participating in the study because they no longer had headaches). Table 1 summarizes patient baseline characteristics (demographic and psychosocial data) and headache diagnoses according to ICDH-II criteria [28] of the 204 participants. Table 2 shows the headache characteristics at baseline of the total cohort and the patient subgroups. 31.9 % of patients were assigned to module 1 (ambulatory), 55.9 % were treated in module 2 (day-clinic) and 12.2 % had in-patient treatment. At admission, migraine patients revealed the lowest headache-related disability (MIDAS: 39.5 ± 35.9), the lowest headache frequency (8.9 ± 4.3 days/month) and the lowest HAD-score for depression (4.34 ± 3.55), while MOH patients had the highest burden of disease (MIDAS: 70.5 ± 73.0), the highest headache frequency (19.6 ± 7.5 days/month), the highest HAD-scores for depression (6.38 ± 3.99) and anxiety (7.83 ± 4.72) and the highest rate of missed school-/workdays per 3 months (35.2 ± 45.8). The group of patients suffering from MOH (33.3 %) consisted of patients with migraine and TTH (51.5 %), migraine (42.6 %), TTH (2.9 %) and others (3.0 %) as the underlying headache disorder. However, in this group 88.2 % reported a withdrawal in medical history and of those, more than half (53.3 %) had experienced more than one withdrawal. In contrast, the group of TTH sufferers had the lowest rate of missed school-/workdays per 3 months (16.3 ± 34.5), the lowest number of days with attack-aborting medication intake (5.1 ± 3.5 days/month) and the lowest HAD-score of anxiety (5.67 ± 5.45). However, implementation of non-pharmacological treatments was lowest in this subgroup at admission (27.3 %), whereas patients suffering from migraine and TTH had the most experience with these techniques (47.5 %).

**Table 1:** Baseline patient characteristics and diagnoses

	Total
Total	204
Age, mean (SD)	42.7 + 13.4
Sex (male/female)	23/181
BMI, mean (SD)	24.1 + 4.6
Education	
Low education level (no or middle school) (%)	123 (65.1)
High education level (high school or higher) (%)	66 (34.9)
Total	189
Marital status	
Single (%)	45 (29.2)
Partnership (%)	92 (59.8)
Others (%)	17 (11.0)
Total	154
Diagnoses	
Migraine (%)	63 (30.9)
Without aura	50 (24.5)
With aura	13 (6.4)
Tension-type headache (%)	11 (5.4)
Episodic	6 (2.9)
Chronic	5 (2.5)
Migraine + tension-type headache (%)	59 (28.9)
Medication-overuse headache (%)	68 (33.3)
Others (%)	3 (1.5)

**Table 2:** Headache characteristics at baseline of the total cohort and the subgroups of patients suffering from migraine, tension-type headache (TTH), migraine and TTH and medication-overuse headache (MOH) and assignement to treatment modules

	Total	Migraine	TTH	Migraine + TTH	MOH
Number of patients	204	62	11	59	69
Duration of disease, Months, mean (SD)	240.4 ± 153.5	229.3 ± 151.7	141.8 ± 118.2	224.9 ± 137.2	279.8 ± 163.7
Headache frequency, days/month (SD)	14.5 ± 8.2	8.9 ± 4.3	15.3 ± 5.5	13.1 ± 7.5	19.6 ± 7.5
Intake frequency of attack-aborting medication, days/month (SD)	10.3 ± 7.3	6.7 ± 3.2	5.1 ± 3.5	6.9 ± 3.9	17.5 ± 7.3
Missed school-/workdays/3 month	24.6 ± 32.3	18.7 ± 18.9	16.3 ± 34.5	19.8 ± 20.7	35.2 ± 45.8
Experience with non-pharmacological treatment (%)	37.9	40.3	27.3	47.5	36.2
MIDAS, mean (SD)	51.94 ± 56.95	39.53 ± 35.89	43.1 ± 79.1	45.55 ± 46.22	70.53 ± 73.00
HADS-depression, mean (SD)	5.29 ± 3.90	4.34 ± 3.55	5.00 ± 5.64	5.29 ± 3.61	6.38 ± 3.99
HADS-anxiety, mean (SD)	7.02 ± 4.16	6.00 ± 3.33	5.67 ± 5.45	7.65 ± 3.93	7.83 ± 4.72
SF 12—physical (SD)	40.75 ± 8.63	41.64 ± 7.74	42.80 ± 9.98	43.02 ± 8.56	38.02 ± 8.45
SF 12—mental (SD)	44.34 ± 10.61	47.09 ± 9.45	46.55 ± 13.94	44.38 ± 10.88	41.67 ± 10.31

### Outcomes

**Fig. 1:**
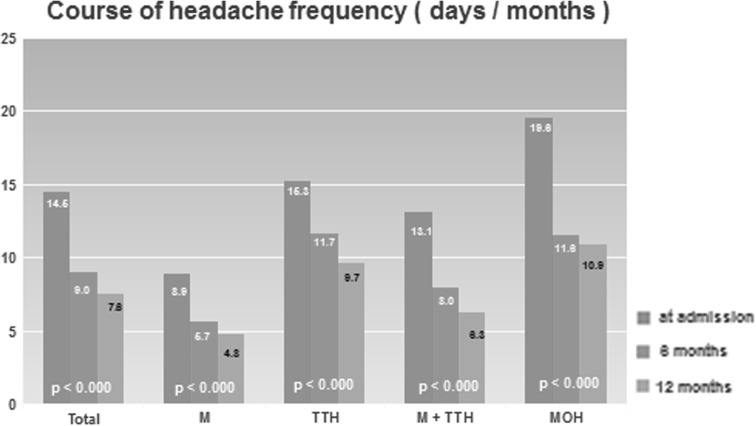
Course of headache frequency (days/month)

The course of headache days per month in the total cohort and the headache subgroups at 6- and 12-month follow-ups compared with baseline headache frequency is shown in Fig. 1. Mean reduction in headache frequency was 5.5 ± 8.5 days per month, *df* = 174, *p* < 0.001 during a 6-month period and 6.9 ± 8.3 days per month, *df* = 194, *p* < 0.001 after 1 year. A reduction in headache frequency of ≥50 % was observed in 128/204 (62.7 %), a reduction of ≥25 % and <50 % in 27/204 (13.2 %), while 40/204 (19.6 %) showed an unchanged frequency (<25 and ≥0 %) and 9/204 (4.4 %) reported an increase of headache days after one year. However, concerning the absolute reduction of days with headache/month, MOH patients improved most (mean −8.7 days per month) starting at the highest level of headache frequency, followed by patients suffering from migraine and TTH (mean −6.8 days per month), while headache frequency decreased least in the group of migraineurs (mean −4.1 days per month), which also had the lowest level of headache frequency at baseline for absolute reduction of days with headache/month. Concerning the relative reduction the decrease was similar among these subgroups. An equally significant reduction of headache frequency could also be observed in all modules after 6 and 12 months. The burden of disease was measured by MIDAS and decreased in the total cohort from 53.0 ± 60.8 points to 37.0 ± 52.4 points, *df* = 107, *p* < 0.001 after 6 months and 34.4 ± 53.2 points, *df* = 154, *p* < 0.001 after 12 months. Missed days at work/school per 3 months were 25.7 ± 35.0 at admission and 17.0 ± 31.9, *df* = 126, *p* < 0.001 at 6 months and 16.8 ± 30.7, *df* = 166, *p* < 0.002 at 12 months. Medication intake of attack-aborting drugs was observed in 199/204 of patients (97.5 %) at baseline and 189/204 (92.6 %) at 12-month follow-up. The intake frequency of attack-aborting medication was 10.3 ± 7.3 days/month at admission, 4.7 ± 4.1 days/month, *df* = 186, *p* < 0.001 after a 6-month period and 3.8 ± 3.5 days/month, *df* = 194, *p* < 0.001 at 1-year follow-up. The highest reduction of medication intake frequency was found in MOH patients (mean −12.3 days/month). A borderline (8–10) or confirmatory (≥11) HAD-score for depression was found in 10.1/6.6 % (total 16.7 %) and for anxiety 25.0/19.0 % (total 44.0 %) of patients. Mean scores for depression/anxiety at baseline were 5.29 ± 3.9/7.02 ± 4.2. Scores for depression/anxiety were highest in the group of MOH patients; mean: 6.

38 ± 3.99/7.83 ± 4.72, and lowest in migraineurs; mean: 4.34 ± 3.55/6.00 ± 3.33. There were no significant differences in depression/anxiety mean scores after 6 months in the total cohort; 5.55 ± 4.44/5.63 ± 4.71, *df* = 63, *p* = 0.982//7.11 ± 4.42/7.03 ± 3.95, *df* = 63, *p* = 0.743. In contrast, mean scores for depression/anxiety showed significant differences after 12 months; mean: 5.29 ± 3.90/5.07 ± 4.36, *df* = 110, *p* = 0.006//7.02 ± 4.16/6.73 ± 4.20, *df* = 110, *p* < 0.001. Use of non-pharmacological evidence-based treatments according the recommendations of the German Headache and Migraine Society [31, 32] was observed in 77/204 (37.9 %) of headache sufferers at baseline and 168/193 (87.0 %) at 12-month follow-up.

Finally, the course of medication-overuse was analyzed during the 1-year follow-up. At baseline, 69/204 patients (33.8 %) had a medication-overuse. At the end of the study, only 3/193 patients (1.6 %) had an overuse of attack-aborting medications with an intake frequency of ≥15 days/month. All of them were medication over-users at baseline; none of the other patients participating in the study developed a medication-overuse within the 1-year follow-up period.

## Discussion

An integrated care program was established to provide multidisciplinary treatment of chronic headache sufferers with frequent migraine, TTH or MOH and/or difficult-to-treat headache. Integrated care at HCB focussed on a comprehensive assessment including a headache diagnosis according to ICDH-II criteria [28], standardized screening for psychiatric comorbidity and provision of treatment according to clinical guidelines [29, 30]. Patients were assigned to three treatment modules following a simple algorithm based on headache frequency, medication use and psychiatric comorbidity. This procedure allowed a valid patient assignment with regard to patients’ headache-related disability and quality of life and tailored treatment to patients’ needs. Recently, this criterion-based assignment for modularized managed care headache treatment has been validated by our study group [40]. A dedicated computer documentation system [35] was introduced to integrated headache care for the first time to enhance the process quality and to realize cross-sectional communication between supply partners and the managed care clinic, as well as online documentation, collection of data from chronic headache patients and risk management.

This study demonstrates that a multidisciplinary in- and outpatient integrated care program is effective in treating chronic headache patients and results in a decrease of burden of disease. Mean reduction in headache frequency was 6.9 days per month at 1-year follow-up. In the present study cohort, a significant difference of headache frequency from baseline to 6- and 12-month follow-ups was observed in all headache subgroups. Harpole et al. [14], treating chronic headache patients in a multidisciplinary management program, reported a reduction of 14.5 headache days on average within 3 months. However, a 3-month follow-up measures mainly short-term effects. Furthermore, in their study, 20 % of the patients had MOH and suffered to 30 % from psychiatric comorbidities, while burden of disease measured by MIDAS was 40.9 points on average. In contrast, an alarming number of 33.8 % patients participating in our study suffered from MOH with a MIDAS-score of 52 points on average, indicating more severely affected patients. A further study by Lemstra et al. [13] reported a reduction of 33.6 % in pain frequency at 3-month follow-up in a small group of migraine patients participating in a 6-week multidisciplinary treatment program. In this study, headaches had existed on average for 101.7 months at baseline (in our study 240 months) and Beck Inventory mean depression levels suggested marked depressed mood levels. Maizels et al. [15] established a group-based model of disease management for patients with headache. During a 6-month period they recognized that severe headache frequency was reduced in 86 % of patients who initially had severe headaches more than 2 days per week. Recently, Gaul et al. [25] reported a reduction of headache frequency of about 36.8 % after 1 year in a large cohort of headache patients treated in a non-modularized multidisciplinary integrated care program. In a 6-month outcome study, Saper et al. [11] assessed that 67 % of mainly physician-referred refractory headache patients who participated in a comprehensive, multidisciplinary, out- and inpatient treatment in a tertiary headache center met the 50 % criterion for both parameters of improvement of headache frequency and frequency of severe headaches. Gaul et al. [24], treating 295 adult patients with a headache-specific multidisciplinary program, and Kabbouche et al. [41], treating children in a comprehensive tertiary care, are the only authors reporting 1-year primary outcome data of multidisciplinary approaches. Headache frequency decreased from 13.4 to 8.8 days/month in adult chronic headache patients, while days with headache/month were 13.4 at baseline and 4.9 after 1 year in children. Jensen et al. [23] reported a reduction of headache frequency from 20 days/month on admission to 11 days/month at the end of treatment after analyzing a total of 1326 patients in a 2-year systematic follow-up study in the Danish Headache Center. In their cohort, 25.5 % of patients had MOH, but unfortunately the authors did not report data indicating burden of disease or psychiatric comorbidity of patients. Furthermore, multidisciplinary integrated care as demonstrated in this study causes a significant reduction of headache-related disability of 18.6 MIDAS points at 12-month follow-up. In the present study, patients with TTH profited most in burden of disease with a reduction of 27.7 MIDAS points, while migraineurs experienced a MIDAS reduction of 11.8 points. This may be due to the fact that in our cohort the TTH subgroup was affected fewest consisting mainly of episodic TTH sufferers (54.5 %), having the shortest history of disease, being fewest anxious and consuming lowest amount of attack-aborting medication/month. Additionally, we observed an absolute reduction of 8.8 lost days at work/school per 3 months. These findings are in accordance with the observations of Harpole et al. [14], who reported a reduction of 21.2 MIDAS points in their study, while Matchar et al. [20] observed just a decrease of 14.9 points. Finally, the present integrated care program was effective in reducing intake frequency of attack-aborting medication (days/month). Intake frequency decreased in the total cohort by about 6.5 days/month and in MOH of about 12.3 days/month. However, medication consumption was examined in just a few studies dealing with multidisciplinary headache treatment. In contrast to our findings, Lemstra et al. [13] investigated a multidisciplinary management program for migraine treatment in comparison with a control group and reported no significant changes in medication use. Furthermore, Maizels et al. [15] studied triptan costs for 6 months before and after intervention using a group-based model of disease management in patients with miscellaneous headaches (mainly transformed migraine with medication overuse). They observed an increase of 19 % in 6-month triptan costs during the interventional phase. On the other hand, observations by Gaul et al. [25] are in accordance with our findings. They described a reduction of acute medication days with intake of analgesics and triptans from 9 to 5 days/month in their multidisciplinary treatment program, which had lifestyle recommendations as an important element in their behavioral treatment concept. Integrated headache care presented in our study focusses likewise on cognitive-behavioral pain management aspects and information about efficacy and possible adverse effects of acute and prophylactic medication and its correct use in headache attack management. Moreover, all patients learned PMR or get another non-pharmacological treatment option in our integrated headache care. The behavioral concept also expected the patients to immediately integrate newly gained knowledge about treating headache into their daily lives. Patients at all treatment levels also received regular instruction to reinforce what they had learned. In particular, cooperating secondary care physicians were requested to provide their headache patients with positive motivation to implement the behavioral changes. Due to this, persistence of medication overuse for one year was documented in only 1.6 % of patients in our study, while at baseline 33.8 % of participants suffered from MOH. This is notable, because in the present study the group of MOH patients was strongly affected, had psychiatric comorbidity in 84 % of cases and prior experience with withdrawal in 88 %; moreover, 53 % of these had multiple withdrawal treatments. The 1-year follow-up outcome after withdrawal of headache medication is assumed in the literature to be up to 40 % [42–47]. However, at the 1-year follow-up, no patients in our study were identified with a newly developed MOH. Epidemiological studies report an incidence for MOH of about 1–4 % [48–52]. In tertiary centers, by contrast, the incidence of MOH reported in studies by Katzarava et al. [53] may even be as high as 14 %. On the other hand only 37.9 % of our patients had experience with non-drug methods of attack relief at baseline. At the end of treatment, the number had risen to 87.0 %, of whom 75 % regularly used PMR. This impressively illustrates that our integrated headache care program results in a long-lasting change in treatment style, away from passive measures and acute medication overuse to an active coping strategy using more non-pharmacological therapies.

A methodological strength of the presented study is its prospective design, the large number of patients, classification of patients according ICHD-II, a comprehensive assessment including measurement of psychiatric comorbidity, implementation of a cross-sector computer documentation system and the long follow-up period of 12 months. The latter especially may help to distinguish between short- and long-term effects. The selected care-research approach may better reflect reality than a controlled study design. But this point also gives rise to a major limitation of our study with a lack of control condition. This non-randomized, open study was conducted at a tertiary headache center taking care of severely affected and chronic headache patients, which may lead to a typical bias. But selection criteria for admission to the integrated headache care could not be influenced by the authors. Future studies should use controlled and randomized design and should clarify the therapeutic role of the different components of treatment in integrated care.

In summary, the present study has provided support for the usefulness of a multidisciplinary integrated care program for severely affected and patients with difficult-to-treat chronic headache, frequent migraine, TTH and MOH. Integrated headache care led to a decrease in anxiety and depression at 12-month follow-up. Further prospective and controlled studies are needed to understand the role of different components of integrated headache care.

## Abbreviations

MIDAS: Migraine Disability Assessment
SF-12: Short Form-12 Health Survey
HADS: Hospital Anxiety and Depression Scale
ICHD-II: International Classification for Headache Disorders-2nd edition
HCB: Headache Centre Berlin
TTH: Tension-type headache
MOH: Medication-overuse headache
